# Unravelling the consequences of the bacteriophages in human samples

**DOI:** 10.1038/s41598-020-63432-7

**Published:** 2020-04-21

**Authors:** Pedro Blanco-Picazo, Dietmar Fernández-Orth, Maryury Brown-Jaque, Elisenda Miró, Paula Espinal, Lorena Rodríguez-Rubio, Maite Muniesa, Ferran Navarro

**Affiliations:** 1 Department of Genetics, Microbiology and Statistics, University of Barcelona, Diagonal 643, Annex, Floor 0, 08028, Barcelona, Spain; 2 European Genome-phenome Archive, Centre for Genomic Regulation (CRG), Doctor Aiguader 88, 08003 Barcelona, Spain; 3 Servei de Microbiologia, Hospital de la Santa Creu i Sant Pau, Institut d’Investigació Biomèdica Sant Pau, Sant Quintí 89, 08041 Barcelona, Spain; 4 Departament de Genètica i de Microbiologia, Universitat Autònoma de Barcelona, Barcelona, Spain

**Keywords:** Bacteriophages, Bacterial infection

## Abstract

Bacteriophages are abundant in human biomes and therefore in human clinical samples. Although this is usually not considered, they might interfere with the recovery of bacterial pathogens at two levels: 1) by propagating in the enrichment cultures used to isolate the infectious agent, causing the lysis of the bacterial host and 2) by the detection of bacterial genes inside the phage capsids that mislead the presence of the bacterial pathogen. To unravel these interferences, human samples (n = 271) were analyzed and infectious phages were observed in 11% of blood culture, 28% of serum, 45% of ascitic fluid, 14% of cerebrospinal fluid and 23% of urine samples. The genetic content of phage particles from a pool of urine and ascitic fluid samples corresponded to bacteriophages infecting different bacterial genera. In addition, many bacterial genes packaged in the phage capsids, including antibiotic resistance genes and 16S rRNA genes, were detected in the viromes. Phage interference can be minimized applying a simple procedure that reduced the content of phages up to 3 logs while maintaining the bacterial load. This method reduced the detection of phage genes avoiding the interference with molecular detection of bacteria and reduced the phage propagation in the cultures, enhancing the recovery of bacteria up to 6 logs.

## Introduction

Bacteriophages (phages), viruses that infect bacteria^1^, are probably the most abundant entities in the world^2^. The abundance of phages in the human body is beginning to be envisaged as having a critical influence on human health. The ability of phage communities to modify and regulate bacterial communities^3,4^ suggests that phages are to some extent responsible for the homeostasis of the microbiota^5,6^.

Phages can contribute to bacterial genomic plasticity by horizontal gene transfer (transduction)^7,8^, which may benefit the metabolism^9^ or affect the virulence of the bacterial host^10,11^. Some phages produce transducing particles consisting of phage capsids that carry only bacterial DNA^12,13^, these transducing particles or elements similar to gene transfer agents (GTAs)^14^ are thought to be mechanisms used by bacterial cells to spread their own genomic content^12^.

The existence of phages in human biomes presupposes their presence in human samples and their contamination of laboratory cultures initiated from these samples^15^. This interference can be envisaged at two levels: 1) phages may propagate in enriched liquid culture media (used to enhance analytical sensitivity and selectively propagate the pathogen) by infecting bacteria (the pathogen targeted for isolation) and causing their lysis during the process; and 2) phages can transport bacterial DNA, including virulence genes such as toxins^16^, antibiotic resistance genes (ARG)^11,17,18^ or bacterial 16S rRNA genes^18^. If any of these genes are targeted by molecular methods, positive results can be a confounding factor in the interpretation of results^19^.

The confirmation of phage interference in microbiological diagnosis, as envisaged in previous studies^15,20^, would represent a serious impediment to the correct detection of bacterial pathogens. It may therefore be useful to evaluate strategies that allow the removal of phages from the human samples without interfering with bacterial recovery or entailing an unacceptable increase in time, efforts and costs. The aim of the present study was to address these issues.

## Results

### Detection of phages in human samples

Phages were detected in all sample types analyzed, first by infectivity assays (Table 1). Between 40–57% of all sample types contained phages able to infect *Escherichia coli* WG5, with the exception of blood (13.5%). Lysis plaques were not observed in any of the other bacterial species tested (*Bacteroides fragilis*, *Enterococcus faecalis* and *Pseudomonas aeruginosa*).

**Table 1:** Human samples in which bacteriophages were detected.

	Ascitic fluid	Blood	Serum	CSF	Urine	Total
N° of samples (individuals)	60	135 (52)	50	56	65 (53)	**366 (271)**
With phages showing infectivity on *E. coli* WG5 (%)*	34 (56.7)	7(13.5)	25 (50)	27(48.2)	21(39.6)	**114 (42.1)**
With phages showing infectivity and observed by TEM (%)	27 (45)	6 (11.5)	14 (28)	8 (14.3)	12 (22.6)	**77 (28.4)**
*% Myoviridae*	0	0	0	0	8.3	**1.5**
*% Siphoviridae*	51.9	50	51.9	12.5	16.7	**41.8**
*% Podoviridae*	18.5	16.7	18.5	37.5	8.3	**20.9**
% Capsid with no tail	29.6	33.3	29.6	25	66.7	**32.8**
*% Inoviridae*	0	0	0	25	0	**3.0**

CSF: Cerebrospinal fluid.

*Only detected with *E. coli* WG5, with *B. fragilis*, *P*. *aeruginosa* and *E*. *faecalis* were not observed.

Later, confirmation of the phage particles obtained from samples showing positive lysis on *E. coli* was conducted by Transmission Electron Microscopy (TEM). TEM observation of phages directly isolated from the samples was performed in those samples containing more than 10^7^–10^8^ phage particles/mL, the minimal required for TEM visualization^13^. Below this concentration no phage particles will be observed. When phages were not so abundant and therefore not observed by direct analysis, they were then recovered from the lysis plaques generated on *E. coli*. Nevertheless, despite our efforts to increase the amount of particles, some samples showing plaques of lysis on *E. coli* did not allow observation by TEM.

The lowest phage detection rate by TEM was in blood samples (11.5%), while serum, *a priori* a sample expected to produce similar results, showed higher percentages in both analyses (infectivity and TEM). On average, infectious phages were observed in 42.1% of the samples and in the 28.4% of them it was possible to visualize phage particles by TEM (Table 1).

**Figure 1:**
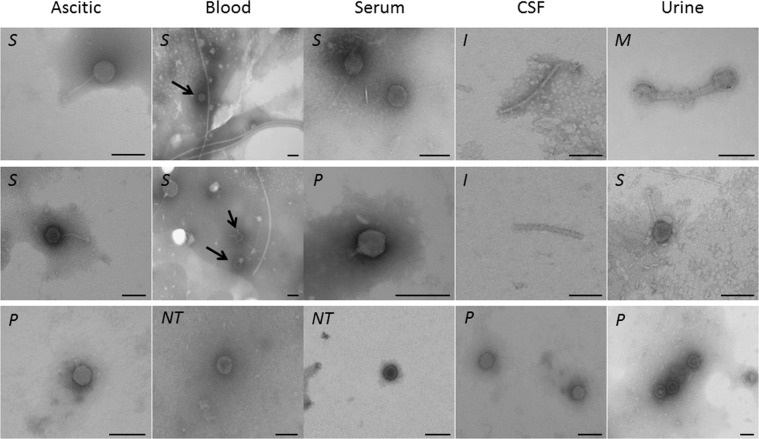
Electron micrographs of phages from ascetic fluid, blood, serum, cerebrospinal fluid (CSF) and urine. (*M*) *Myoviridae*, (*S*) *Siphoviridae*, (*P*) *Podoviridae*, (*I*) *Inoviridae* and (NT) structures compatible with phage capsids but not showing a tail. Bar 100 nm.

*Myoviridae*, *Siphoviridae* (the most frequent), and *Podoviridae*^21^ phage morphological types were observed. Many samples showed icosahedral capsids of 40–60 nm of diameter compatible with these three groups but without a tail, which made them indistinguishable (Fig. 1). In two cerebrospinal fluid (CSF) samples (25%), filaments compatible with phages of the *Inoviridae* morphological type were observed (Table 1).

### Virome analysis

**Figure 2:**
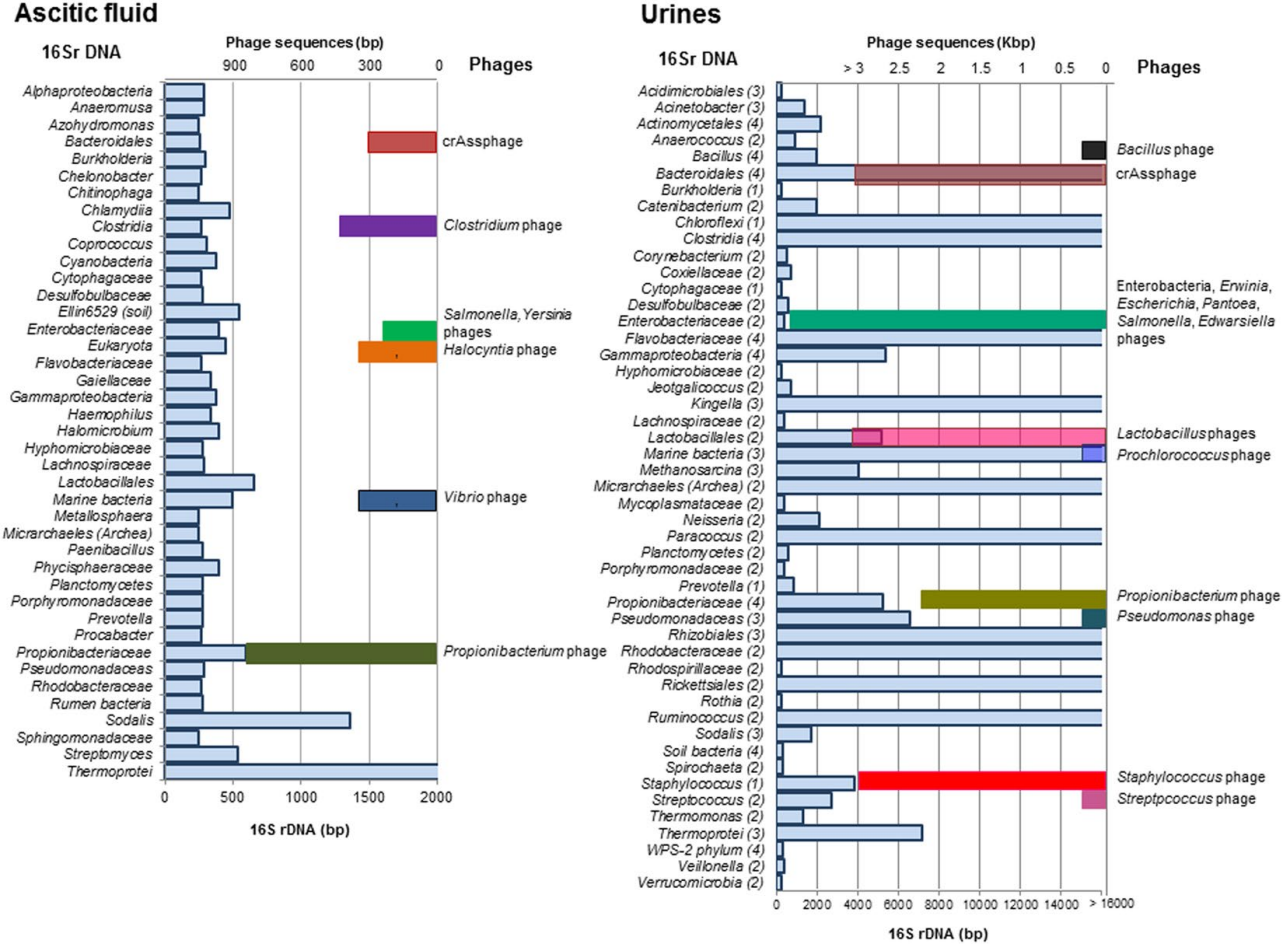
Identification of the 16S rRNA gene sequences found in the virome of a pool of ascitic fluids, and four pools of urine samples. On the left side of the chart: 16S rRNA gene sequences of diverse bacterial genera identified in the viromes. On the right side of the chart, bars indicate the coincidence with sequences of bacteriophages infecting particular bacterial groups. The length of the bars corresponds to the length of the contig in base pairs (bp). In the urine samples, next to each bacterial group and in brackets is shown the number of urine viromes in which the 16S rRNA gene sequence of each genus was detected.

Four pools of urine samples and one pool of ascitic fluid (AF) samples allowed the recovery of viral DNA in sufficient quantity and purity to generate the libraries. Before the capsids were broken, the samples were tested for 16S rRNA genes, and negative results confirmed the absence of non-packaged DNA and the effectiveness of the protocol^18^. Analysis of the virome showed a great number of unclassified sequences, that was greater in the AF and in two urine pool samples (Table 2), but the abundance of unclassified sequences did not correlate with the number of phages detected. The viromes confirmed the presence of bacteriophages in the samples and revealed coincidences with phages infecting different bacterial genera (Table 2; Fig. 2). Even if the identification of phage sequences by Kraken suggests a possible bacterial host, this cannot be confirmed only by sequence comparison with the databases. Nevertheless, all samples showed sequences of phages coincident with phages infecting *Propionibacterium* and *Staphylococcus*, and all samples showed the presence of CrAssphage, a human-specific phage first detected *in silico*^22^ that infects the genus *Bacteroides*. Viruses other than bacteriophages were detected in the viromes, being a poxvirus (BeAn 58058 virus)^23^, human endogenous retrovirus and polyomaviruses the most frequently found in ascetic fluid. The latter ones, papillomaviruses and adenoviruses were the most frequently detected in the urine viromes.

**Table 2:** Viromes of the human samples and bacterial genes detected.

Name of Virome	Nº of samples in the pool	Virome size: Nº of reads (Nº nucleotides)	Nº of contigs (Nº nucleotides)	Nª of classified contigs (%)	Nº of unclassified contigs (%)	Nº of different phages	Possible phage host (number of phages)*
12	15 ascitic fluids	8456720 (2016.48 Mbp)	187729 (54.41 Mbp)	2774 (1.48%)	184955 (98.52%)	35	*Aeromonas* (1), *Bacillus* (2), *Caulobacter* (1), *Clostridium* (1), *Halocyntia* (1), *Propionibacteium* (22), *Salmonella* (1), *Staphylococcus* (1), *Streptococcus* (1), *Vibrio* (2), *Yersinia* (1), crAssphage** (1)
14	17 urines	50078697 (13973.96 Mbp)	170329 (60.57 Mbp)	15491 (9.09%)	154838 (90.91%)	64	*Brochothrix* (1), *Enterobacteria* (1), *Erwinia* (3), *Escherichia* (7) *Pantoea* (1), *Salmonella* (5), *Enterococcus* (1), *Mycobacterium* (1), *Prochlorococcus* (1), *Propionibacterium* (36), *Pseudomonas* (1), *Staphylococcus* (4), *Streptococcus* (1), crAssphage (1)
15	21 urines	3516412 (999.81 Mbp)	35005 (14.99 Mbp)	17188 (49.10%)	17817 (50.90%)	47	*Aeromonas* (1), *Bacillus* (1), *Enterococcus* (2), *Lactobacillus* (4), *Lactococcus* (1), *Listeria* (1), *Mycobacterium* (1), *Propionibacterium* (29), *Rhizobium* (1), *Staphylococcus* (1), *Streptococcus* (5), crAssphage (1)
16	15 urines	4867915 (1359.94 Mbp)	30101 (20.00 Mbp)	22117 (73.48%)	7984 (26.52%)	44	*Enterobacteria* (1) *Edwardsiella* (1), *Escherichia* (3), *Salmonella* (1), *Enterococcus* (1), *Lactobacillus* (4), *Propionibacteium* (30), *Staphylococcus* (2), crAssphage (1)
17	6 urines	14093699 (3501.02 Mbp)	131414 (51.45 Mbp)	5362 (4.08%)	126052 (95.92%)	29	*Bacillus* (1), *Bacteroides* (1), *Enterococcus* (1), *Lactobacillus* (3), *Propionibacterium* (18), *Staphylococcus* (4), crAssphage (1)

*Number of different phages detected in each virome that show homology with sequences in the databases of phages infecting the bacterial host described.

**crAssphage is a particular type of human-specific phage infecting *Bacteroides* genera.

*Siphoviridae* was the most abundant morphological type identified in phageomes, followed by *Myoviridae*. Virome 12 showed 89% of *Siphoviridae* and 9% of *Myoviridae*; virome 14 showed 87.5% *Siphoviridae* and 7.8% *Myoviridae*, virome 15 shows 85.1% *Siphoviridae* and 6.4% *Myoviridae*, virome 16 showed 88.6% *Siphoviridae* and 9.1% *Myoviridae* and virome 17 showed 75.9% of *Siphoviridae* and 13.8% *Myoviridae*. There was a lower presence of *Podoviridae* and unclassified morphology that corresponded mostly to CrAssphage viruses.

### Bacterial genes in the virome

The five viromes analyzed (12, 14–17) showed bacterial DNA in the unassembled reads and in the contigs. While the bacterial DNA contamination during the viral DNA extraction was excluded by the controls used, the possibility of bacterial DNA contamination during the sequencing process was also excluded by the negative samples. Moreover, human sequences were not found in our viromes as should be expectable in case of contamination. Considering all this, we concluded that the bacterial DNA identified should be located within the phage capsids. We observed a great diversity of 16S rRNA gene sequences from different bacterial groups in contigs of different lengths (from 250 bp to various hundreds of bp) (Table 3; Fig. 2). Comparison of viromes of the four urine samples revealed that they shared some bacterial 16S rRNA gene sequences. Some bacterial genera to which this 16S rRNA gene belonged matched the hosts of the bacteriophages detected in the samples (Fig. 2, black bars). Other phages did not match the 16S rRNA gene sequences found in the viromes, for example, *Aeromonas*, *Bacillus* or *Caulobacter* phages in the AF, and *Brocothirx*, *Prochlorococcus*, *Listeria*, *Mycobacterium* and *Enterococcus* phages in urines.

**Table 3:** Bacterial genes detected in the virome of the samples.

Virome	N° of 16S rRNA gene*	N° ARG*	Antibiotic and resistance genes (bp)	ARG flanking region
12	78	1	Tetracyclines*: tetC* (1013)	P: *Salmonella*
14	96	15	Aminoglycosides: *aph(3”)-Ib* (804)*, aadA5* (789)*, aph(6)-Id* (831)	P: *E. coli* or *Klebsiella*
			β-lactams: *bla*_OXA-396_ (789), *bla*_TEM-1B_ (860), *bla*_PAO_ (1194)	C: *Pseudomonas*
			Fosfomycin: *fosa* (408)	C: *Pseudomonas*
			Macrolides: *mdfA* (1233)*, mphA* (906)	P: *E. coli*
			Quinolones: *crpP* (198)	C: *Pseudomonas*
			Sulfonamides: *sul1* (840)*, sul2* (816)	P: *E. coli*
			Tetracyclines: *tetC* (923)*, tetA* (1200)	P: *Salmonella* or *Vibrio*
			Trimethoprim: *dfrA17* (474)	P: *E. coli*
15	45	6	Aminoglycosides: *aadA1* (758)*, aadA2* (792)	P: *E. coli* or *Klebsiella*
			β-lactams: *bla*_TEM-116_ (861)	C: *Propionibacterium* or *Mycoplasma*
			Phenicols: *cmlA1* (1260)	P: *E. coli* or *Klebsiella*
			Sulfonamides: *sul3* (792)	P: *E. coli*, C: *Salmonella*
			Tetracyclines: *tetA* (1200)	P: *E. coli*
16	37	14	Aminoglycosides: *aac(3)-IIa* (861)*, aph(6)-Id* (796)*, aph(3”)-Ib* (521)	P: *E. coli*. C: *Klebsiella* or *Rheinheimera*
			β-lactams: *bla*_PAO_ (1182), *bla*_CTX-M-28_ (540), *bla*_TEM-1B_ (733)	C: *Pseudomonas* or *Klebsiella*. P: *E. coli*
			Fosfomycin: *catB7* (522)*, catB3* (442)	C: *Pseudomonas*
			Quinolones*: oqxA* (1151)*,qnrB1* (645)*, fosA* (366)	C: *Klebsiella*, *Citrobacter* or *Salmonella*
			Tetracyclines: *tetA* (1015)	P: *E. coli*, *Klebsiella*, *Salmonella*, *Shigella*
			Trimethoprim: *dfrA14* (474)	P: *Klebsiella*
17	73	10	Aminoglycosides: *aph(3’)-III* (795)*, ant(6)-Ia* (909)*, aac(6’)-Ii* (549)	PH: *Streptococcus* phage C: *Enterococcus*
			Macrolides: *mdfA* (1233)*, msrC* (1479)*, ermB* (738)*, ermT* (544)	PH: *Streptococcus* phage C: *Enterococcus*
			Tetracyclines*: tetM* (1225)*, tetL* (1343)	C: *Enterococcus*, *Staphylococcus*
			Trimethoprim: *dfrG* (498)	C: *Enterococcus*

P: Plasmid, C: Chromosome, PH: Phage genome

*Number of different 16S rRNA genes or different ARGs that were detected in each virome as verified by Kraken.

The viromes also contained a diverse range of ARGs, conferring resistance to aminoglycosides, β-lactams, fosfomycin, macrolides, phenicols, quinolones, sulfonamides, tetracyclines or trimethoprim (Table 3). Only those genes with a minimal identity of 97% in at least 60% of the sequence were defined as ARGs. The analysis of ARG flanking showed that most ARGs were located in bacterial genomes (plasmids or chromosome), and only one virome (#17) was found to contain genes conferring resistance to aminoglycosides (*aph(3*′*)-III* and *ant(6)-Ia*) and to macrolides (*ermB*) located in a *Streptococcus* phage genome (GenBank: KT336321.1), although these genes also blasted against the *Enterococcus* chromosome.

### Interference of phages with the molecular detection of bacteria

**Figure 3:**
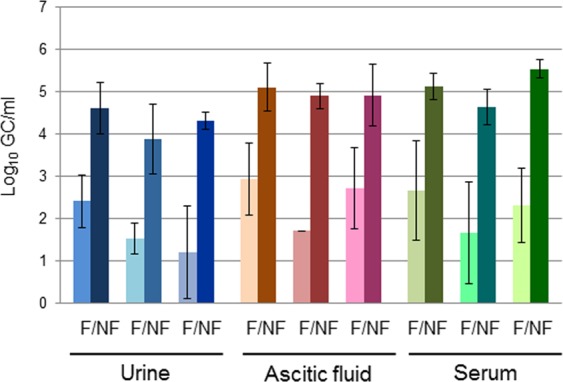
Phage reduction (log_10_ gene copy [GC]) of the Stx phage after filtration through 0.45 μm low-protein-binding polyvinylidene fluoride membranes (F) versus non-filtered samples (NF). The results correspond to three independent experiments on each matrix.

The elimination of phages from the samples was explored with the aim of finding a methodology that allows phage reduction without disturbing bacterial recovery, a methodology that is suitable for routine use and has a minimal cost. Phages were removed physically by membrane filtration using DURAPORE membranes of 0.45 μm pore size. These low-protein-binding membranes allowed phage particles to pass through while retaining bacteria. To verify that phages had been physically removed from the samples, first a specific qPCR was performed targeting a Stx phage. Urine, AF and serum samples were spiked with 10^4^ Stx phage particles (assuming that each phage carries one *stx* copy, the GC (gene copy) value is equivalent to the number of phages carrying *stx*) (Fig. 3). After filtration, a significant (p < 0.05) reduction from 2.17 to 3.22 log_10_ units in the number of Stx phages was observed in all the samples analyzed and in all three independent replicates (Fig. 3).

### Bacterial strain recovery

**Figure 4:**
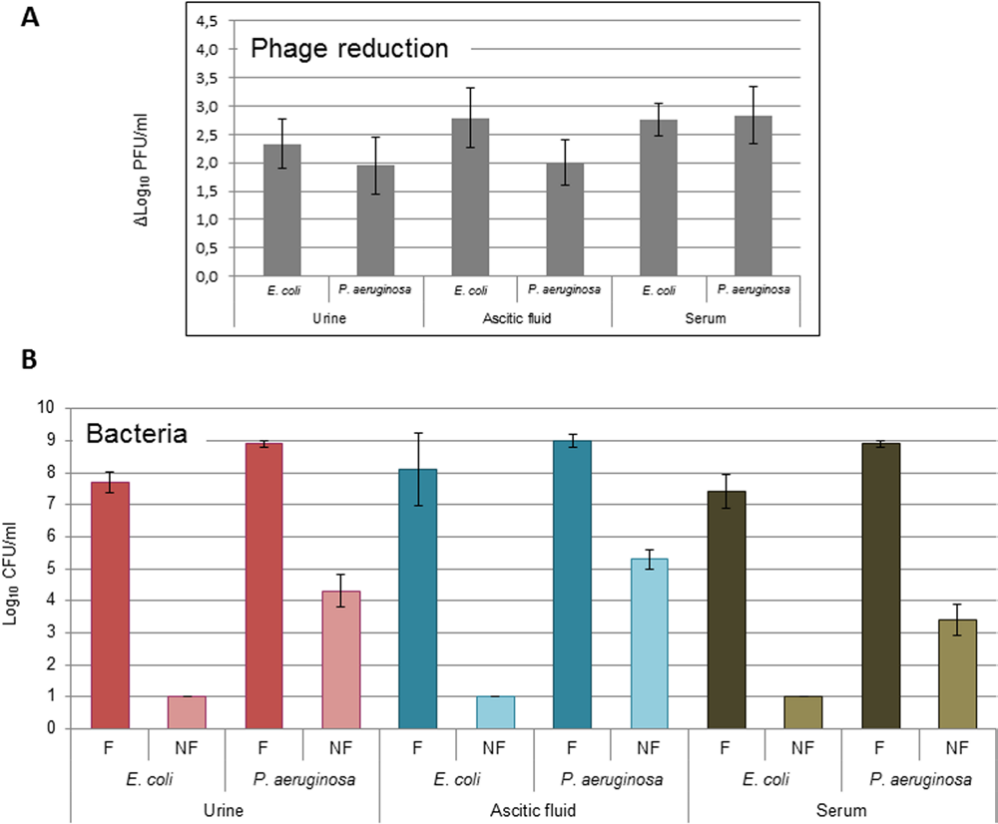
Phages can bias the outcome of enrichment cultures. (**A**) Reduction (log_10_ PFU/mL) of phages infecting *E. coli* and *P. aeruginosa* in urine, AF and serum samples after the filtration procedure. (**B**) Recovery of culturable *E. coli* and *P. aeruginosa* (log_10_ CFU/mL) in enrichment cultures of urine, AF and serum samples with (F) and without (NF) the filtration procedure. The results shown are the average of three independent experiments and error bars indicate the standard deviation.

Urine, AF and serum samples containing *E. coli* or *P. aeruginosa* and their respective phages were processed with and without filtration. The influence of the filtration method on bacterial recovery was evaluated by comparing samples on an enrichment culture incubated for 18 h at 37 °C before and after processing (Table 4, Fig. 4).

**Table 4:** Values of phage (PFU/mL) and bacteria (CFU/mL) in the samples after the application of the filtration method (F) in comparison with the non-filtered controls (NF).

		*E. coli*			*P. aeruginosa*		
		Urine	Ascitic Fluid	Serum	Urine	Ascitic Fluid	Serum
Average phage counts	F	6.50 × 10^2^ (1.58 × 10^2^)	1.60 × 10^2^ (1.89 × 10^2^)	2.33 × 10^2^ (1.44 × 10^2^)	1.63 × 10^2^ (7.77 × 10^1^)	6.67 × 10^1^ (2.89 × 10^1^)	6.33 × 10^1^ (1.58 × 10^1^)
	NF	1.03 × 10^5^ (1.00 × 10^4^)	1.18 × 10^5^ (6.66 × 10^3^)	1.63 × 10^5^ (8.96 × 10^3^)	7.10 × 10^4^ (1.95 × 10^4^)	6.42 × 10^4^ (1.74 × 10^4^)	4.33 × 10^4^ (7.57 × 10^3^)
Average bacterial counts	F	7.67 × 10^1^ (4.04 × 10^1^)	4.63 × 10^2^ (3.09 × 10^1^)	1.75 × 10^2^ (1.06 × 10^1^)	8.00 × 10^1^ (8.66 × 10^1^)	1.57 × 10^2^ (1.00 × 10^2^)	1.07 × 10^2^ (3.66 × 10^1^)
	NF	9.50 × 10^1^ (8.66 × 10^0^)	2.25 × 10^2^ (9.57 × 10^0^)	1.83 × 10^2^ (7.64 × 10^0^)	1.17 × 10^2^ (2.89 × 10^1^)	3.37 × 10^2^ (1.04 × 10^2^)	1.13 × 10^2^ (4.54 × 10^1^)

In brackets, standard deviation.

A significant reduction of 2–3 log_10_ PFU/mL was achieved for both *E. coli* and *P. aeruginosa* phages (Table 4, Fig. 4A) without affecting the bacterial recovery (Table 4). After incubation, the bacterial counts in filtered (F) samples were significantly (p < 0.05) higher than in the non-filtered (NF) samples (Fig. 4B). *E. coli* showed greater differences between F and NF samples than *P. aeruginosa*, suggesting higher rates of coliphage propagation in *E. coli* strain WG5. A difference in recovery was not attributable to the different matrices.

## Discussion

In a previous study by our group, infectious bacteriophages were found in a limited set of urine and AF samples, resulting in phage interference with the isolation of the bacterial pathogen^15^. In the current study, a greater abundance of phages was detected in a wider range of human samples, indicating the presence of phages not only in urine and AF, but also in blood-sera and CSF with variable prevalence. While the detection of phages in animal serum samples was reported some time ago^24^, and confirmed by metagenomics of blood^25^, to our knowledge this is the first report of infectious phages in CSF of humans. A recent study of CSF in patients receiving hematopoietic stem cell transplantation showed contigs mapped to phages^26^, although it is not clear whether the phages were already present in the patients, acquired after transplantation, or indeed if the CSF contained phages or remnant phage DNA.

The lowest percentage of phage detection was in blood samples, whereas nearly 50% of serum samples contained infectious phages. Although serum and blood samples are essentially the same, the blood samples were diluted in blood culture bottles prior to analysis, whereas sera were not. Moreover, blood is more difficult to handle, particularly for filtration through the membranes, which became clogged due to the high density of the samples. When blood was analyzed by TEM, a large number of particles of undefined nature were visible, which interfered with phage visualization and identification. As only clearly recognizable phage capsids were taken into account, a large number of phages in the blood probably remained undetected. In contrast, serum samples were more concentrated and easier to handle than blood, and phage prevalence detected in sera was markedly higher. The *Siphoviridae* morphological type was the most common group, as previously reported^15,18,27^. *Siphoviridae* detection could have been underestimated in a percentage of samples showing icosahedral phage capsids compatible with *Siphoviridae* morphology but without a tail, which prevented their correct classification^18,28–30^.

The wide range of phage hosts restricts their detection by culture. For example despite detecting phages of *Bacteroides*, *Enterococcus* or *Pseudomonas* in the viromes, phages infecting some strains of these bacterial genera have not been detected in this study. Moreover, phage detection by microscopy is limited by the density of phage particles (since TEM requires a minimum of 10^7–8^ phage particles). In contrast, a more detailed picture of the diversity of phages in the samples was provided by the virome analysis. The multi-step phage DNA purification protocol was previously validated to guarantee that only DNA within viral capsids was evaluated^18^. This procedure, together with the limited number of phages in some of the samples, meant that sufficient DNA concentration and purity for the metagenomic analysis was only obtained in five pools of samples. Nevertheless, the viromes analyzed showed a far greater abundance of phages infecting a wider range of bacterial genera than in our previous study^18^. Phage identification was based on Kraken results, which in turn were based on database sequences. Therefore, the homology with an entry of a phage infecting a particular bacterial genus does not confirm that the bacterial host of this phage has been identified; it only indicates a potential host. Virus-host assignment is one of the most challenging issues when working with metaviromes and results obtained should be only considered as a match with the information in the databases. Genome similarity between different phages^31^ and the limited sequence data available for phages may have resulted in an underestimation or misidentification of phages, as a fraction of contigs were not classified. Surprisingly, some phage sequences in our viromes matched with phages infecting environmental bacteria. Moreover, the 16S rRNA genes of these bacterial groups have also been detected. Although unexpected, marine bacteria, *Rizhobium* or plant pathogens have previously been reported in gut, urine or ascitic metagenomes^32–36^. Accordingly, the detection of phage sequences matching with phages infecting these bacterial groups becomes more reasonable. These bacterial species and their phages could have been acquired through the diet or by cross-contamination between human biomes (e.g fecal-ascites, fecal-urine). It may also happen that there is an incorrect identification caused by similar viral sequences in the databases.

Viruses other than phages detected in the viromes were not as diverse or as abundant as phages, but this could be caused by the absence of infectious viruses in the samples if there was no viral infection and/or by the lower densities of animal viruses in comparison with the densities of bacteriophages, as previously reported^30,37^.

As in the case of serum and blood, the inherently sterile CSF and AF could have been expected to give similar results. Yet considerable differences were observed, with AF giving the highest percentage of positive samples and CSF the lowest after blood. This indicates that translocation of bacteria and/or phages may occur between the digestive tract and peritoneal fluid, even spontaneously, and to a greater extent in patients with ascites. This translocation may be easier for virus particles than for bacteria^15,37^. On the contrary, CSF is protected by the meninges and is located further away from highly contaminated regions such as the digestive tract.

In the case of urine, classically considered as sterile, it is exposed to contamination by the flora of the distal urethra, vagina and/or perineum during collection. Whether the phages in urine samples proceed from the urinary tract or the collection process, their presence can interfere with bacteriological analysis.

The presence of phages in a human sample has further implications when bacterial detection is based on targeting a specific gene, for instance, eubacterial 16S rRNA genes. Phages incorporate bacterial genes through lateral^12^, generalized^7^ and specialized transduction^7,37^. Regardless of the mechanism, phage particles packaging 16S rRNA genes have been reported in fecal phageomes^18^. Since DNA extraction methods do not distinguish between phage and bacterial DNA^38^, the presence of phage DNA complicates pathogen identification or produces false positives. In this study, the diversity of 16S rRNA gene sequences in phage particles greatly exceeded the range of identified phages (Fig. 2), which may be explained by the higher number of bacterial 16S rRNA gene sequences in the genomic databases in comparison with the short repertoire of available phage genomes.

The bacterial genes identified in the viromes included ARGs, which are among the most relevant genes mobilized in phage capsids. The implications of this mobilization go beyond incorrect molecular detection, as it confirms that phage particles, by acting as vehicles for gene transfer and transduction, are a mechanism for the spread of ARGs^39–41^. The ARG flanking regions corroborated that phage particles contained mostly bacterial and only in a few cases phage DNA^13,18,42^.

The size of phages allows them to be removed easily by relatively cost-efficient protocols. In the present study, membrane filtration reduced phage particles without disturbing bacterial recovery and favored bacterial isolation by impeding phage propagation and bacterial lysis. These results confirm previous observations, in which *E. coli* was isolated from agar plates from AF samples containing phages, but not after enrichment in liquid cultures^15^.

Phage abundance in human samples is highly variable, so it is not always a key factor in bacterial recovery and/or identification. However, when abundant, phages can clearly interfere with diagnosis at different levels. The occurrence of phages in the human body was unsuspected until relatively recently. Now that their prevalence in human biomes has been demonstrated, this knowledge should be reflected in microbiological diagnosis by the incorporation of suitable protocols.

## Methods

### Samples

A total of 366 samples from 271 individuals were analyzed: 135 blood culture vials (referred to henceforth as blood) from 52 individuals, 65 urine samples from 53 individuals, 60 ascitic fluid (AF) samples from 60 individuals, 56 cerebrospinal fluid (CSF) samples from 56 individuals and 50 serums from 50 individuals. All samples were obtained from the Microbiology Laboratory of the Hospital de la Santa Creu i Sant Pau (Barcelona, Spain) and were used after performing a conventional microbiological diagnosis.

All methods were carried out in accordance with official ethics regulations (Biomedical Research Law 14/2007, of July 3rd, and the provisions of Royal Decree 1716/2011, of 18th November) (http://www.eurecnet.org/legislation/spain.html), which establishes the treatment of biological samples of human origin for biomedical research purposes. The procedures were approved by the Clinical Research Ethics Committee (CEIC) of the Biomedical Research Institute of the Hospital de la Santa Creu i Sant Pau (IIB-Sant Pau), Barcelona, Spain. Approval of the Ethics Committee included the exemption of obtaining the informed consent of the participants considering that samples were completely anonymized, that no data on the patients were collected and that samples were not stored but were destroyed immediately after the study.

### Detection of phages in the samples

5 mL of each sample was used for phage purification as previously described^15^. If the volume was insufficient (as in the case of CSF), it was raised to 2 mL with phosphate buffer saline (PBS). Samples were filtered through low protein-binding 0.22-µm-pore-size membrane filters (Millex-GP, Millipore, Bedford, MA), treated with chloroform (1:10), vortexed for 2 min and centrifuged at 16 000*xg* for 5 min, and then the supernatant was collected.

Phages were evaluated for infectivity using strains *Escherichia coli* WG5^43^, *Bacteroides fragilis* RYC2056^44^, *Enterococcus faecalis* ATCC 29212 and *Pseudomonas aeruginosa* 9108 as hosts by the double agar layer method^1^.

### Transmission electron microscopy (TEM) studies

10 µl of phage suspensions were dropped onto copper grids with carbon-coated Formvar films, negatively stained with 2% ammonium molybdate (pH 6.8) and examined under a Jeol 1010 transmission electron microscope (JEOL Inc. Peabody, MA) operating at 80 kV.

### Virome analysis

Four pools of urine samples and one pool of AF samples (comprising between 6 and 21 samples) (Table 2) selected among those that showed presence of bacteriophages in the previous experiments were pooled to obtain viral DNA in sufficient quantity and purity to generate the libraries. Viral DNA was extracted as previously described^18^. Briefly, samples were filtered by 0.22-µm-pore-size membrane filters (Millex-GP, Millipore), 20-fold concentrated using protein concentrators, chloroform-treated to break possible vesicles and digested with DNase I (100 units/mL; Sigma-Aldrich, Spain) for 1 hour at 37 °C to eliminate non-packaged DNA. The DNase was inactivated by heating the suspension at 70 °C for 10 minutes. The absence of non-packaged bacterial DNA was verified by real time qPCR amplification of eubacterial 16S rRNA gene^13,18^. These controls are expected to be negative if the protocol successfully eliminates most of the vesicles and DNA outside the viral capsids. Controls were applied to verify absence of inhibitors and correct DNase inactivation as in previous studies^18,45^. Briefly, after DNAse inactivation 10^3^ GC of *bla*_TEM_ gene were added to the controls and amplified by qPCR. An incomplete DNase inactivation, or the presence of inhibitors, would have resulted in a lower number of *bla*_TEM_ GC which was not observed.

Packaged DNA was extracted using a PowerSoil® DNA isolation kit (Qiagen Iberia, Barcelona. Spain).

Sequencing was performed as previously described using Illumina libraries generated following the Nextera XT (Illumina, Inc., San Diego, CA, US) manufacturer’s protocol for paired-end libraries (2 × 150 bp)^18^. Bioinformatic analysis to identify phages in the virome and bacterial genes was performed as previously described^18^.

Sequence reads were quality checked with FASTQC v0.11.2 to detect any anomalies in the sequencing process. Raw data obtained from sequencing were processed with Trimmomatic (v0.36)^46^. Clean reads were acquired by removing low-quality reads, sequences with N ratio >3%, and adapter sequences in reads. Reads were then *de novo* assembled with default parameters (k-mer sizes 21, 33, 55, 77 and 91) using SPAdes v3.13^47^. Contigs were classified taxonomically with Kraken v0.10.5^48^. ARGs were searched with ResFinder 3.2^49^, with a 90% ID and 60% minimum length threshold. Sequences flanking the ARG genes detected by Resfinder were examined with BLAST^50^ to ascertain whether the sequences were phage or bacterial. The search for 16S rDNA sequences was performed with Kraken2 with GreenGenes supported database (13_8) taking into account the contigs generated by Spades. To exclude contamination from the reagents used for metagenomic analysis^51^, negative controls were performed using negative samples that were processed in parallel and used the same DNA extraction protocol than the viromes presented in this study.

### Removal of phages from the sample

To evaluate phage removal and bacterial recovery, urine, ascitic fluid and serum samples were spiked with 10^2^ CFU/mL of *E. coli* strain WG5 or *P. aeruginosa* strain PA14 and with 10^5^ PFU/mL of phages that infect each strain (isolated from sewage). The spiked samples were divided in 2 aliquots of 10 mL, one remained untreated and the other was filtered through 0.45 μm low-protein-binding polyvinylidene fluoride (PVDF) membranes (DURAPORE membrane filter, Millipore) using a vacuum system that required from 1–5 minutes with the volumes of sample filtered in this study. Filtration step allowed bacteriophages to pass through the membrane while retaining bacteria. To remove more phages, the membranes were washed twice with 10 mL of PBS, gently agitated, and the filtration was repeated. The other 10 mL-aliquot was processed without filtration.

Bacteria retained in the filter after the filtration procedure were recovered by resuspending the membrane in 10 mL of PBS. The homogenate was added to 10 mL of Luria Broth (LB) 2-fold concentrated. In parallel, the non-filtered aliquot (10 mL) was also added to 10 mL of LB 2×. Bacteria and phages were enumerated in both aliquots by growth on LB agar plates (CFU/mL) and a double agar layer^1^, respectively. Then, filtered (F) and non-filtered (NF) aliquots were incubated at 37 °C for 18 h and bacteria were enumerated on LB agar plates after incubation.

Molecular confirmation of phage reduction by filtration in urine, AF and serum samples was achieved by inoculating a suspension containing 10^5^ PFU/mL of Stx phage (containing the Shiga toxin (*stx*_2_) gene), and the number of phages was evaluated by qPCR analysis of the *stx*_*2*_ gene^52^.

### qPCR procedures

qPCR assays for *stx* and 16S rRNA genes were performed as previously described^18,52^ under standard conditions in a Step One RT PCR System (Applied Biosystems). Genes were amplified in a 20 μl reaction mixture with the TaqMan Environmental Master Mix 2.0 (Thermo Fisher Scientific. Waltham, MA). The reaction contained 1 μl of the DNA sample or quantified plasmid DNA^18,52^. All samples were run in triplicate, as well as the standards and negative controls. Gene copy number (GC) was defined as the mean of the triplicate data obtained.

### Statistical analysis

Statistical tests were performed using one-way analysis of variance (ANOVA) in the Statistical Package for Social Science software (SPSS v19). A *p* < 0.05 was used to evaluate the differences between filtered versus non-filtered samples.

## Data Availability

The metagenomic data set generated was deposited to BioProject (PRJNA590575). Data can be checked under the following link: https://dataview.ncbi.nlm.nih.gov/object/PRJNA590575?reviewer=g6bbejub0bdrr64b5ir5jkti3. The URL will expire when this BioProject is publicly-released. All data generated are available from the corresponding author on reasonable request.
